# Bavarian Universities

**Published:** 1837-10

**Authors:** 


### BAVARIAN UNIVERSITIES.

[We are indebted to a friend now travelling in Germany for the following short account of the regulations respecting students generally, and medical students more particularly, in the universities of Bavaria.]

The Professors of the German Universities are for the most part, if not all, appointed by government, which also lays down the rules for the management of the University. This interference of Government with the internal affairs of the University is found in different degrees in different countries. To take Bavaria and Austria for example:—in the former, the professors are left at liberty to conduct their own departments as they choose; in the latter, on the contrary, even the books employed must be prescribed by government. From all this it will be perceived that the Universities possess no such thing as *charters of privileges.*

1. *Of Matriculation.* Matriculation is an essential preliminary for the admission of students. For this purpose there is a Matriculation Committee, composed of the Rector of the University, the ministerial Commissary and the head of the police. Matriculation commences for the Winter Session on the 19th October, for the Summer Session, on the Monday after Easter. The matriculation books are open fourteen days at the most. Matriculation confers on the student the Academical burger-right, the right of residence at the University, of attendance on the teachers, and making use of all the means of instruction which the University offers.

2. *Of the Studies.* Every native, who comes to the university with the intention of preparing himself for a public office, for which a complete course of University study is required, is obliged, during his attendance at the university, in case he has not previously been at a Lyceum, to apply himself with zeal and industry, as well to the study of the general sciences, as to the study of the particular sciences of his future calling; and for this a period of five years is necessary, unless he can show that, at the end of the fourth year of his University course, he is in every respect perfectly prepared. In this case, the medical student can be admitted at the end of the fourth year of study to the *examen pro gradu.* In the medical *examen pro gradu,* proofs are required of a knowledge of natural science, chemistry, anthropology, and psychology; and, whoever has not certificates of attendance on these subjects, must have certificates from one or more members of the Faculty, testifying that he is known to them as a diligent and industrious student. It is allowed for natives who have passed from the gymnasium to the university, even at the first period of their university residence, to attend lectures introductory to their own department in addition to those on the general sciences: thus, students in medicine may attend general anatomy, comparative anatomy, general chemistry, pharmacy, natural history of the three kingdoms, and physiology, which are reckoned as introductory to the study of medicine. Candidates, however, are required, at the end of the first year, or at the latest at the end of the second, to undergo a strict public examination in the general departments, such as logic, general history, philology, mathematics, as also natural history and physics.

3. *Extent of the Courses.* The harvest vacation lasts from the 1st September to the 18th October, and the Easter vacation from the beginning of passion-week to the Monday after Easter week: the sessions, therefore, last about five months each. There is no interruption to the lectures except on Sundays and authorized holidays. Students must be punctual in being present at the commencement, and continue their attendance closely until the termination of the session. The student must enter his name for each of the courses of lectures he means to attend, immediately, or at the latest nine days after the commencement.

4. *Of the Discipline.* German students compose a separate *caste* of the community. They are under distinct laws and amenable to the jurisdiction of distinct officers: it is the University police alone which has power over them. On their admission, students receive a certificate from the university police, which must be renewed every session. This the student is required always to have by him, "in order that he may be able to prove by it, in every case, his connexion with the university; and he has himself to blame, if he, from neglect of this, be regarded and treated as if he were not a student." No student may, without leave from the rector, be more than one night absent from the university. Students in medicine or surgery who are present at a duel, as medical men, are visited with the same punishment as seconds. All students, on admission, must subscribe an obligation not to connect themselves with any political or other unauthorized society.

5. *Of the Fees.* The professor's honoraria are from nine to fourteen florins, (15*s.* to 24*s.*); and the degree comes to about sixty florins, (56*l.*)

---

